# Wolves contribute to disease control in a multi-host system

**DOI:** 10.1038/s41598-019-44148-9

**Published:** 2019-05-28

**Authors:** E. Tanner, A. White, P. Acevedo, A. Balseiro, J. Marcos, C. Gortázar

**Affiliations:** 1 Maxwell Institute for Mathematical Sciences, Department of Mathematics, Heriot-Watt University, Edinburgh, EH14 4AS UK; 2 SaBio, Instituto de Investigación en Recursos Cinegéticos IREC (CSIC-UCLM), Ciudad Real, Spain; 3 SERIDA, Gobierno del Principado de Asturias, Gijón, Spain; 4 Animal Health Department, University of León, León, Spain; 5 Gobierno del Principado de Asturias, Oviedo, Spain

**Keywords:** Ecological modelling, Population dynamics, Ecological epidemiology

## Abstract

We combine model results with field data for a system of wolves (*Canis lupus*) that prey on wild boar (*Sus scrofa*), a wildlife reservoir of tuberculosis, to examine how predation may contribute to disease control in multi-host systems. Results show that predation can lead to a marked reduction in the prevalence of infection without leading to a reduction in host population density since mortality due to predation can be compensated by a reduction in disease induced mortality. A key finding therefore is that a population that harbours a virulent infection can be regulated at a similar density by disease at high prevalence or by predation at low prevalence. Predators may therefore provide a key ecosystem service which should be recognised when considering human-carnivore conflicts and the conservation and re-establishment of carnivore populations.

## Introduction

Infectious agents that can be transmitted to more than one host species form the majority of pathogens that infect wildlife, domestic and human systems^1^. Wildlife species play a key role in maintaining reservoirs of infection^2^ and therefore disease management requires strategies to reduce transmission of pathogens from wildlife reservoirs to target hosts^1^. It has been shown that predation may contribute to disease control in multi-host systems leading to reduced spillover to livestock and human populations^3,4^. Therefore predators can provide a key ecosystem service that is often underappreciated by society^5,6^.

Mathematical models have played a key role in uncovering the potential of predators to control zoonotic disease where theory has shown that predators may act to alter the epidemiological dynamics to decrease infected and increase susceptible host density and thereby reduce prevalence^4,7,8^. Furthermore, selective predation on infected individuals can reduce the force of infection and in extreme scenarios prevent pathogen establishment^9,10^. However, model analysis has also outlined scenarios in which predation may lead to an increase in disease prevalence – notably when the disease induces a long-lasting immune response^11^. This highlights the importance of understanding the case-specific infection dynamics of pathogens in reservoir populations that are subject to predation. Empirical evidence to underpin the theory on the interplay between predation and host infection is however limited. Hudson *et al*.^12^ suggested that macroparasite incidence in grouse (*Lagopus lagopus scotica*) populations decreased when predator levels increased and Levi *et al*.^13^ showed that increases in the incidence of Lyme disease correlated with a decline in small mammal predators. More recently, observational and experimental studies have indicated that parasites can increase host susceptibility to predation^8,14^ (see^15^ for a recent review). Therefore combining theory and empirical data at the system specific level has the potential to further clarify the role of predation in the control of infectious disease reservoirs in wildlife^6^. We investigate this by combining model results with field data for the case study system of wolves (*Canis lupus*) that prey on wild boar (*Sus scrofa*), a reservoir of tuberculosis, in Asturias in northern Spain.

Animal tuberculosis (TB), caused by infection with *Mycobacterium bovis* and closely related members of the *M*. *tuberculosis* complex (MTC), is a widespread multi-host infection with a profile of moderately increasing prevalence among cattle herds in infected regions of western Europe (from 1.05% in 2010 to 1.49% herd prevalence in 2015^16^). TB causes severe economic losses to the livestock industry due to movement restrictions and compulsory test and slaughter schemes^17,18^. TB also causes host mortality^19^ and creates conservation concerns due to potential spillover to endangered species (e.g. to Iberian lynx)^20,21^. The role of wildlife reservoirs in maintaining TB is now well recognised with reservoir species including cervids in North America, European badgers (*Meles meles*) in the British Isles, brushtail possums (*Trichosurus vulpecula*) in New Zealand and buffalo (*Syncerus caffer*) in South Africa, among others^18,22^. In Europe, and in particular on the Iberian Peninsula, infection is maintained in a complex network of domestic and wild hosts, including abundant wild ungulates such as the Eurasian wild boar which acts as the primary reservoir of infection^18,23,24^.

In multi-host settings, TB control at the wildlife-livestock interface often targets aspects such as direct and indirect contacts between host species^25–27^ and TB control in reservoir hosts^28^. It has been shown that culling of wild boar can reduce TB prevalence in wild boar and sympatric host species^29,30^. However, the role of ecosystem functioning in regulating infection transmission has not been assessed in detail. The wolf is the most widely distributed top predator of the northern hemisphere^31,32^ where wild boar and deer are its main prey^33,34^ and wolf presence has been linked with lower ungulate prey densities^5^. It has also been found that when wolf populations decrease, wild boar populations tend to increase^35,36^ (but see^37^). Mathematical modelling studies have suggested that wolves may contribute to disease control in their prey in the case of Chronic Wasting Disease in North American deer (*Odocoileus sp*)^10^. Moreover, empirical evidence suggested that anthrax infection in bison (*Bison bison*) might increase wolf predation risk^38^. It has also been suggested that pathogens targeting the lung may predispose ungulate prey to wolf predation^39,40^. Hence, maintaining viable wolf populations might contribute to disease control in wildlife and thereby reduce transmission from wildlife reservoirs.

Asturias, in north-western Spain, is an area with an established wolf population that occupies two-thirds of the region^41^. TB is present in Asturias although the current overall prevalence in wild boar (2–13%) and the level of generalised cases (17% from tests on 6 infected individuals) are lower than in TB-endemic regions of southern Spain where TB prevalence can be >50% (with 80% prevalence reported in some regions^42^) and where a greater proportion (58%) of infected individuals are generalised^43^. Here we distinguish between individuals that are infected with TB (but not infectious) and those with generalised infection that can infect other individuals through direct contact and that shed infectious particles. Generalised individuals also suffer disease-induced mortality and their poor health increases their vulnerability to predation. Asturias is also a cattle-breeding region, with 360,735 heads in 16,312 herds in 2014 and TB is one of the main concerns of cattle farmers^44,45^. However, the potential role of wolf predation as a natural regulator of disease in wild ungulates is not widely recognised by farmers^46^. Asturias can therefore be used as a case study region in which to test the impact of wolf predation on TB prevalence in a wildlife reservoir species (wild boar) and on TB control in the target species (cattle).

In this study we combine field observations from Asturias with mathematical modelling to test the hypothesis that TB prevalence is reduced in the presence of wolves compared to when wolves are absent. Moreover, the model findings allow us to explore the long-term impact of predation on TB control, to explain how compensatory population growth may result from a reduction in disease-induced mortality due to predation and as a consequence explain how information on prevalence and population density is necessary to assess the risk of spillover from wildlife reservoirs. The results provide important insights into the role predators can play in disease control and therefore inform on the debate related to human-carnivore conflicts and the conservation and re-establishment of carnivore populations^5,6,47,48^.

## Results

### Wolf population

The annual number of reported wolf attacks on livestock increased from 1481 in 2000 to 3024 in 2014 (100% increase; Supplementary Information Fig. S1). Reports of wolf predation on livestock were unrelated to livestock numbers. Instead they correlated positively to the number of wolf packs and to wolves culled during the previous season^49^. Therefore we extrapolate wolf numbers from these wolf attack data using linear regression to ascertain the linear growth rate of the wolf population over this period (Fig. S1). Using the data on wolf numbers for 2003–2004 as 252^50^, we estimate the number of wolves in 2000 as 196 growing linearly to 392 in 2014.

### Wild boar population

**Figure 1:**
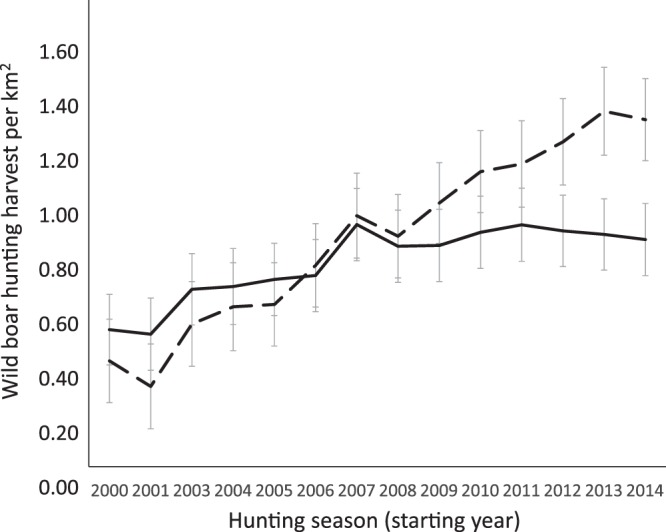
The mean annual wild boar hunting harvest/km^2^ in Asturias, for the period 2000–2014. The dotted line represents areas where wolves are absent and the solid line those where wolves are present. Bars represent 95% confidence intervals.

Areas with and without wolves had similar wild boar harvest rates in year 2000 (0.52 and 0.40 wild boar/km^2^, respectively). By 2014 harvest rates had increased to 0.85 in areas with wolves but had a greater increase to 1.32 in areas without wolves. Between 2008 and 2014, the wild boar hunting harvest grew steadily in areas without wolves but remained stable in areas with wolves. Specific hunting effort data is not available for the areas with and without wolves, however there is no known difference in the type of hunter typical to these areas as in Asturias as a whole, hunting is non-commercial and traditional among rural inhabitants^51^. The use of hunting statistics as a proxy for wild boar abundance trends is well-established in ungulates^51–54^. By this method we assume that wild boar density was 50% higher in areas without wolves than areas with wolves (Fig. 1). Therefore in the areas with wolves we estimate wild boar density as 1.65/km^2^ in 2000 rising to 2.55/km^2^ in 2014. In the areas without wolves we estimate wild boar density as 1.2/km^2^ in 2000 rising to 3.6/km^2^ in 2014.

### TB prevalence

A total of 1051 wild boar sera were tested for antibodies against MTC, yielding a mean seroprevalence of 5.42% (95% CI 4.21–6.98) for the whole study period. The reduction in seroprevalence between periods was significant in sites with wolves (the southern more mountainous regions) where prevalence declined by 77% from 16.67% ± 7.47% in 2000–2007 to 3.87% ± 1.76% in 2008–2014 (Fisher’s p < 0.0001). In sites without wolves prevalence was initially lower and no significant change in prevalence was recorded: 6.89% ± 10% in 2000–2007 and 3.08% ± 3.5% in 2008–2014 (Fig. S2). The mean annual cattle herd TB prevalence from 2005 to 2007 was 0.19%. Herd prevalence grew slightly in 2008–2014, reaching a mean of 0.22%. In areas with wolves, cattle TB herd prevalence remained almost stable during the study period (0.22% in 2005–2007; 0.19% in 2008–2014; with Yates Chi^2^ = 0.45, 1 d.f., p = 0.5 indicating no significant difference between the prevalence levels). By contrast, in areas without wolves herd prevalence increased by 56% in the same period: 0.16% in 2005–2007; 0.25% in 2008–2014; with Yates Chi^2^ = 7.18, 1 d.f., p = 0.0074 indicating that the difference between prevalence levels is significant (Fig. S2).

### The model comparison to data for regions with wolves

**Figure 2:**
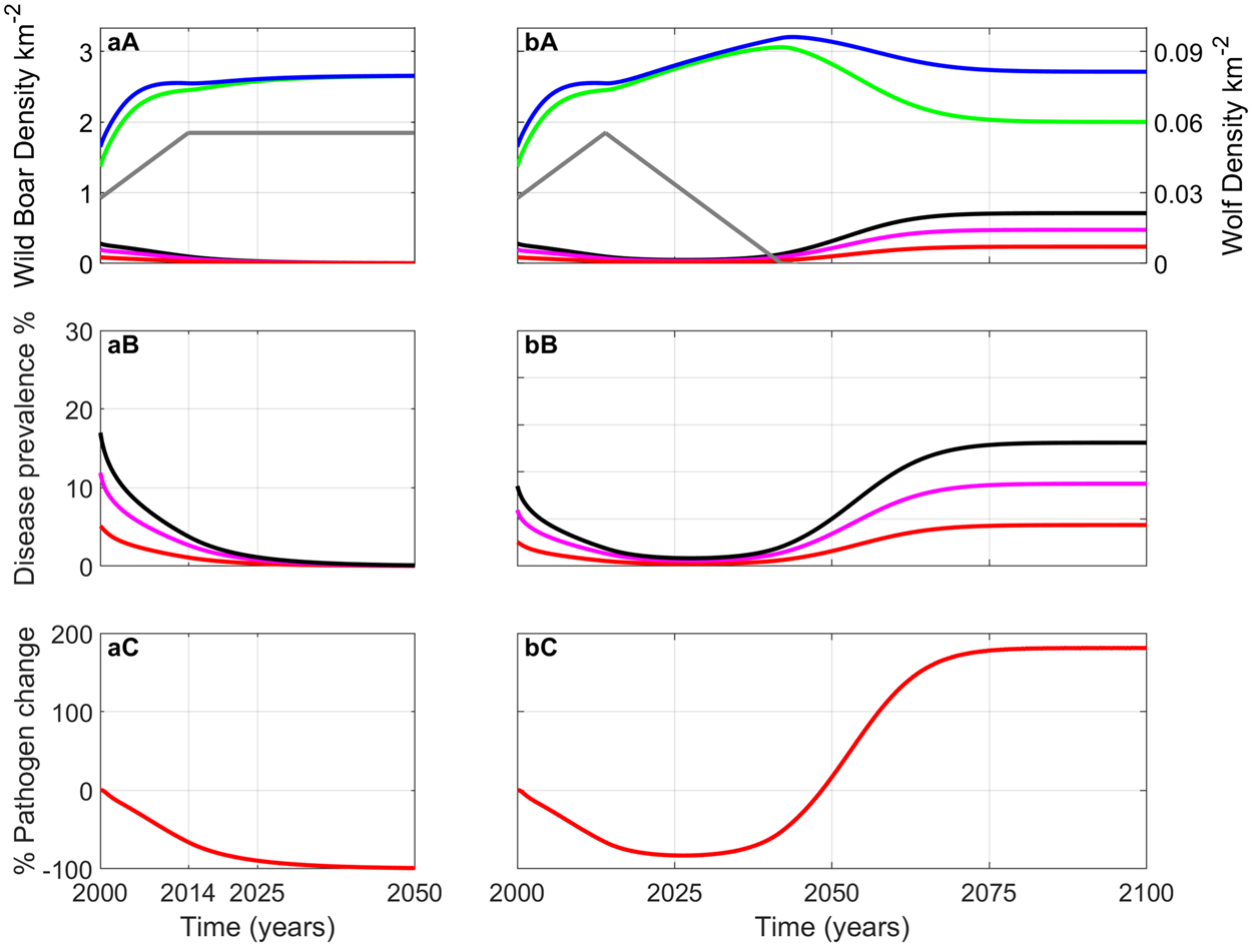
Model results for the sub-region of Asturias inhabited by wolves. (**a**) Wolf numbers rise from 196 (2000) to 392 (2014) and then remain constant until 2050. (**b**) Wolf numbers rise from 196 (2000) to 392 (2014) and then decrease at the rate at which they increased until they die out. Initial conditions set wild boar and wolf densities to their 2000 values taken from the field data, and the initial prevalence ($(I+G)$/*N*) in 2000 is 17% (of which 29% are generalised). (**A**) changes in wild boar population density - total population (blue); total susceptible (green); total infected and generalised (black); infected (magenta); generalised (red); wolves (grey). (**B**) changes in total prevalence (black), infected prevalence (magenta) generalised prevalence (red). (**C**) % change in the density of environmental pathogen. For parameters see Supplementary Information.

The model results for the wild boar population density, TB prevalence and the percentage change in the level of pathogen in the environment in response to a linear increase in wolf density are shown in Fig. 2 for the period 2000–2014. As wolf density increases there is a decrease in TB prevalence from 17% in 2000 to 3.8% in 2014. This highlights that predation by wolves could be a key factor in reducing TB prevalence in wild boar. The level of generalised infection remains relatively constant at 29% of the total infected population throughout the study period. The reduction in prevalence leads to a more than 50% reduction in the level of pathogen in the environment by 2014. Model results indicate that wild boar density increased from 1.65/km^2^ to 2.55/km^2^ between 2000 and 2014 with the density starting to saturate from 2008. This is in close agreement with the observed data.

A key finding is that although wolf numbers increase, which will increase overall predation, there is also an increase in wild boar density. This increase in wild boar density can be attributed to an assumption that wild boar were below their carrying capacity in 2000 and so positive growth would be expected, but also because predation decreases TB prevalence and therefore decreases the population level mortality due to TB. Hence, the increased mortality due to predation is compensated by a reduced TB induced mortality. An implication of the approximately two-fold increase in wild boar population density and four-fold decrease in prevalence is that the level of pathogen in the environment decreases by more than 50% over the 14-year period. This is significant since a reduction in the free-living particles reduces the risk of infection in other animals, in particular livestock, which share the same environment as the wild boar.

The pronounced reduction in TB prevalence (from 17% in 2000 to 3.8% in 2014) assumes selective predation by wolves on wild boar piglets and generalised individuals. In comparison (Supplementary Information and Fig. S3), if wolves prey indiscriminately on all wild boar classes the prevalence reduction is 17% to 8.3% but the wild boar density only grows to 2.10/km^2^ in 2007 before declining to 1.93/km^2^ in 2014. If wolves prey on piglets only prevalence shows a reduction from 17 to 9.5% over the 2000 to 2014 period (Fig. S4). The model results therefore suggest that predation on generalised individuals is key to the significant reduction of prevalence since the removal of generalised individuals reduces infection from both direct contact and environmental contamination.

### The impact of wolves on TB prevalence in the long-term

We examine the long-term impact of predation by wolves on TB prevalence in wild boar for different trends of wolf density (Fig. 2). In Fig. 2a we assume wolf numbers remain constant after 2014 (reflecting that wild boar are a key component of wolf diet). There is a small increase in wild boar density due to reduced disease-induced mortality as a consequence of the further reduction in TB prevalence, but in general, predation by wolves is sufficient to stabilise wild boar numbers. TB prevalence and the level of environmental pathogen decrease to low levels. This emphasises how predation can control virulent infection in a prey species and also reduce the risk of infection to other host species. In Fig. 2b we assume wolf density will decrease and reach zero in 2042. This represents a scenario where wolves are intentionally removed. Here, as wolf numbers initially decrease there is a rise in wild boar density with TB prevalence in wild boar remaining low. However, as wolf numbers decrease further TB prevalence increases leading to a downturn in wild boar density in response to increased disease induced mortality. It is notable that the final stable wild boar numbers in the absence of wolves (Fig. 2b) are similar to the level in the presence of wolves (Fig. 2a). However, a key difference is that TB prevalence is low (0.1%) in the presence of wolves and high (26%) in their absence. This has significant consequences for potential environmental transmission of MTC from wild boar to other species. The underlying mechanism responsible for this difference is that wild boar density is largely regulated by the disease in the absence of wolves whereas it is regulated by predation in their presence. This is a key insight from the mathematical model. It highlights how restrictions to predator growth may have only minor impacts on prey density but a major detrimental impact on the prevalence of infection in prey species.

### Model comparison to data in areas of Asturias without wolves

**Figure 3:**
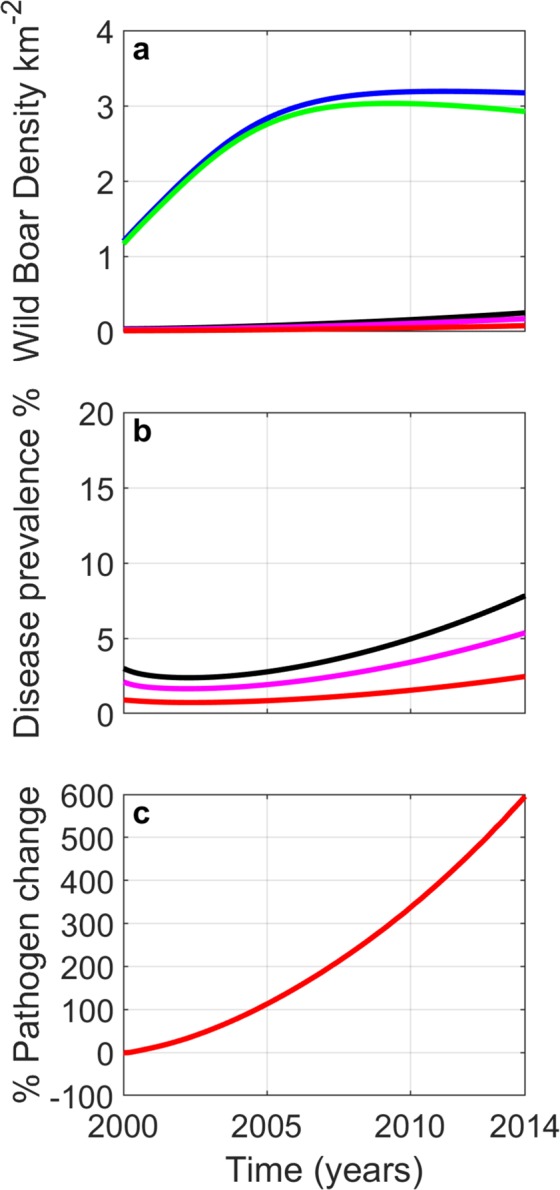
Model results for the sub-region of Asturias not inhabited by wolves. Initial conditions set wild boar and wolf densities to their 2000 values, and the initial prevalence ($(I+G)$/*N*) in 2000 is 3% (of which 30% are generalised). (**a**) Changes in wild boar population density - total population (blue); total susceptible (green); total infected and generalised (black); infected (magenta); generalised (red); wolves (grey). (**b**) Changes in total prevalence ($(I+G)$/*N*) (black); infected prevalence (*I*/*N*) (magenta); generalised prevalence (*G*/*N*) (red). (**c**) % change in the density of environmental pathogen. For parameters see Supplementary Information.

The results for the model that reflect the region of Asturias in which wolves are absent are shown in Fig. 3. Here, there is a rapid increase in wild boar density, with close to a 3-fold increase in density between 2000 and 2014 (which reflects the increase in density observed in the field data). TB prevalence initially remains constant (at around 3%) but from 2007 onwards shows an increasing trend reaching a prevalence of 7.8% by 2014. This relatively low increase in prevalence coupled with a large increase in population density leads to a large increase (over 500%) in the level of environmental pathogen and therefore a potentially increased risk of infection spillover to co-habiting domestic and wild animals.

### The potential impact of predation in regions of high TB prevalence in wild boar

To represent areas of high TB prevalence we modify the baseline parameters for Asturias to reflect increased prevalence and generalised infection. In such regions wild boar density is typically high (due to management and artificial feeding) even though environmental conditions are harsh and in particular severely diminished water availability necessitates the sharing of water holes and leads to overall poor body condition^55^. This increases the level of environmental transmission and leads to a more rapid transition from the infected to the generalised class for piglets and yearlings^28^ (see also Supplementary Information). We assume here that wild boar live at an endemic density 8/km^2^, and adjust *K* and *q* to reflect this (see Supplementary Information). Other parameters remain as in the set-up in Asturias and in particular note that to maintain the comparison with Asturias we do not change the background culling rate. In the absence of wolves the model results indicate a prevalence of 57% of which around 54% are individuals with generalised infection (this is in good agreement with Muñoz-Mendoza *et al*.^43^). In Fig. S5 we introduce wolves at a constant density of 0.08/km^2^ which represents an initial wolf to wild boar ratio of 1:100. Initial predation by wolves reduces wild boar density, but primarily affects infected and generalised individuals. This causes a reduction in TB prevalence and therefore reduced population level disease-induced mortality. This drives an increase in susceptible individuals and an increase in wild boar population density which promotes a resurgence in disease prevalence. Infection and population recovery oscillates until after 50 years the population has increased wild boar numbers (10.1/km^2^), reduced TB prevalence to 26.5% and reduced levels of environmental pathogen by 54%.

**Figure 4:**
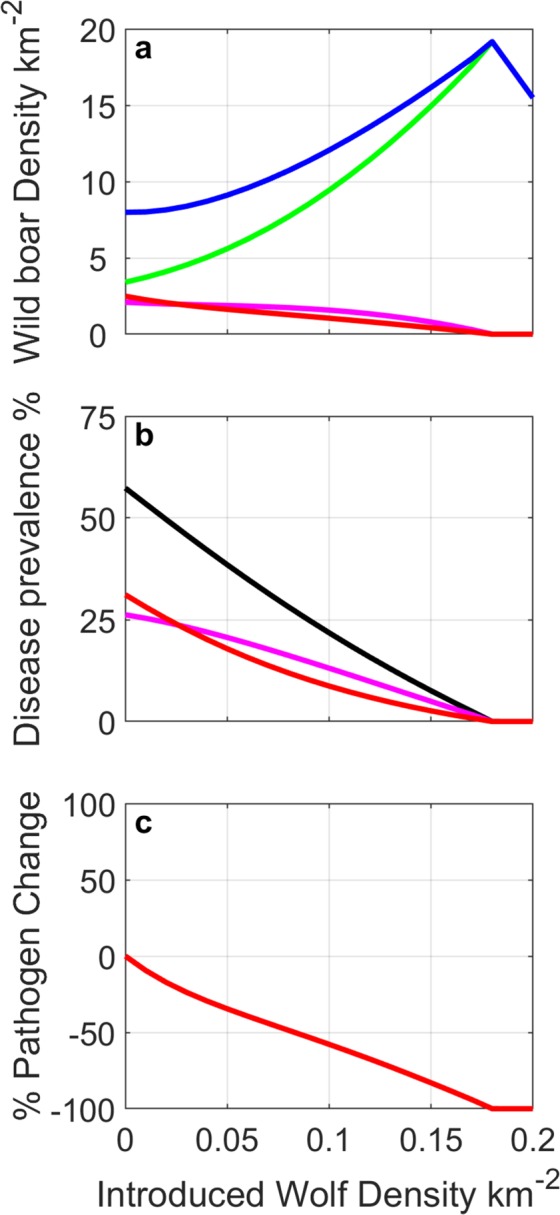
Model results for areas with high TB prevalence showing the long-term outcome after different constant densities of wolves are introduced to a wild boar population with density steady at 8/km^2^ and disease prevalence ($(I+G)$/*N*) of 57%. (**a**) Changes in wild boar population density - total population (blue); total susceptible (green); infected (magenta); and generalised (red). (**b**) Changes in total prevalence ($(I+G)$/*N*) (black); infected prevalence (*I*/*N*) (magenta) generalised prevalence (*G*/*N*) (red). (**c**) % change in the density of environmental pathogen. For parameters see Supplementary Information.

Figure 4 shows the impact of wolf density on the steady state level of wild boar density, disease prevalence and environmental contamination. In the absence of wolves the model results indicate a prevalence of 57% of which around 54% are individuals with generalised infection (this is in good agreement with^43^ for wild boar TB prevalence in Mediterranean Spain). As wolf numbers increase the level of disease prevalence and risk of environmental contamination decrease. However, the density of wild boar increases as wolf density (and predation) increases. This increase in wild boar density is a direct result of the decrease in TB prevalence as the mortality from predation is lower than disease induced mortality due to TB that was experienced in the absence of wolves. There is a threshold in wolf density that leads to disease eradication and for wolf densities above this threshold there is a decrease in wild boar density (since mortality from predation is no longer compensated following eradication of the disease).

## Discussion

In this study we combine field data and theory for a case study system to confirm the hypothesis that the presence of wolves can lead to a reduction in TB prevalence compared to when they are absent. Our findings indicate that wolf predation may contribute to TB control in wild boar, reducing TB prevalence and the release of MTC into the environment. These factors are likely to contribute to reduced levels of indirect transmission from the wild boar infection reservoir to other hosts. The results have wide-ranging implications that highlight how predation can play a key role in the control of infectious disease in multi-host systems.

It has been postulated that MTC transmission between wild and domestic hosts is mostly indirect, mediated by contaminated vegetation, water, mud, feed or other substrates^18,55^. Wild boar are the primary reservoir host for MTC in Spain with infection to other host species likely to be through indirect transmission in regions where multiple hosts overlap^56^. Wild boar are relatively long-lived^57^ and older age classes can mount a formidable defence against predation. Therefore wolves are likely to select generalised (severely infected) individuals (which are the class responsible for shedding pathogen to the environment^55^) or piglets (which is an age group more likely to suffer generalised infection^58^). Such selective predation has been suggested as a key mechanism which can decrease infection prevalence in prey^3^ and was shown to lead to reduced prevalence of prion disease in cervids without a dramatic decrease in their density^10^. Our field observations and model study show that there is a reduction in wild boar disease prevalence without a consequent reduction in wild boar density in regions where wolves might selectively target piglets and generalised wild boar. Our results indicate that the decrease in prevalence would be less pronounced if predation targeted all classes indiscriminately or if it targeted only piglets. Therefore, our results support previous findings^3,10^ that suggest the ability of predators to preferentially select the most infected prey may be key to their role in disease control. Moreover, our findings suggest that wolves could play a key role in TB control in wildlife reservoirs in Spain. In Asturias, the annual cost of compensation paid to farmers due to wolf attacks on their livestock (€1,016,860) is a quarter of the annual expenses of the cattle TB eradication scheme (€4,163,348; Regional Government 2014). The ecosystem service provided by predators in terms of disease control should form part of the debate when discussing the impact of predators since here wolves may be allies of farmers, rather than enemies.

In the absence of wolves (Fig. 3), wild boar numbers increase significantly. Model results indicate that there is a lag between the increase in wild boar growth and the increase in TB prevalence since the increase in infected individuals has a similar increasing trend to that of the overall population. This could explain the observation that TB prevalence in wild boar in the absence of wolves has remained relatively fixed. Note, however, that while TB prevalence in wild boar has remained constant the model predicts that the density of generalised wild boar and the presence of MTC in the environment increases throughout the study period. It is notable that the empirical findings for areas of Asturias in which wolves are absent show that there is a near five-fold increase in TB detected in cattle between 2000–2014. Our model provides an explanation for how a small percentage increase in prevalence coupled with a large increase in population density in a reservoir population may lead to a large increase in environmental contamination. This could explain the observed increase in cattle TB in these regions.

The model system was adapted to examine the potential impact of predation on disease control beyond the Asturias case study system (Fig. 4). In areas with high TB prevalence such as central and southern Spain, the observed prevalence of TB is 50% and an increased proportion of those infected exhibit generalised infection (58%). Since predators may select the most severely infected individuals there is the potential for predation to have a greater impact on disease control in such settings. More specifically, as there is a higher prevalence of generalised individuals, there will proportionately be more predation on these super-shedders and therefore the potential to have an exaggerated effect on removing the wild boar that are responsible for shedding the pathogen in the environment, thus having greater potential to reduce spillover to other wild and domestic hosts. In this scenario our model results show that predation by wolves does lead to an exaggerated reduction in disease prevalence while leading to an increase in overall population density and reduction in the level of environmental pathogen. This increase in wild boar density is a direct result of the decrease in TB prevalence as the mortality from predation is lower than disease induced mortality due to TB that was experienced in the absence of wolves. This emphasises the generality of our findings and further highlights the potential role of predators in disease control.

Previous theoretical studies that have shown that, in disease regulated populations, predation can reduce the force of infection and thereby decrease the density of infected hosts, increase the density of susceptible hosts and lead to an increase in overall population density^3,10^. Our model study shows that increased mortality from predation is approximately balanced by a reduction in disease-induced mortality. A key result is therefore that the prey population can be regulated by the disease, with consequent high prevalence in the prey species or at a similar density by a predator but with low disease prevalence. This finding highlights how restrictions to predator growth may have only minor impacts on prey density but a major detrimental impact on the prevalence of infection in prey species. The mechanism that underlies the compensatory balance between predation and disease induced mortality has recently been explained in systems subject to culling/harvesting^59^. Tanner *et al*.^59^ show that in systems that lack long-lived immunity to infection, population reductions from harvesting are compensated due to a population level release from disease-induced mortality. The compensatory effect increases as disease virulence increases and occurs for systems with density-dependent, frequency-dependent and environmental (free-living) modes of transmission. They explain how harvesting in systems that harbour virulent parasites can lower disease prevalence without significantly reducing, or indeed can increase population density. Our findings show how mortality from predation is compensated by a release from disease-induced mortality that can reduce TB prevalence and the potential spillover of infection to sympatric hosts^29,30^. Tanner *et al*.^59^ also show that in systems in which individuals develop long-lasting immunity following infection harvesting leads to a significant reduction in population density and an increase in infected prevalence and agree with theory that examines the impact of predation in systems with long-lasting immunity^11^. This highlights the necessity to understand the system specific host infection dynamics that are subject to predation or harvesting^59–61^.

Our results agree with earlier findings that the removal of a predator from a system that is regulated by both predator-prey interactions and virulent infection may increase disease prevalence and suppress prey abundance^3,12,13^. Our model results suggest that in the initial years of wolf removal wild boar density can increase and disease prevalence stays low. This may indicate that predator removal can be beneficial, however this is only a transitory state. When the wolf reaches sufficiently low numbers the disease is able to re-infect the increased abundance of susceptibles so that over time the population becomes regulated by disease rather than predation. This is accompanied by an increase in environmental contamination and risk of spillover to other wild and domestic hosts. This further highlights the complexity and potential negative consequences of predator removal and the need to consider disease status in predator management programmes.

Our modelling results show good agreement with the field data for our case study system. We expect our general findings relating to reduced prevalence of TB and compensatory growth of wild boar in the face of predation to be robust to changes in our model assumptions. The key requirement is that TB is virulent and individuals do not recover to develop long-lasting immunity^59^. There are however specific aspects where the model and field study disagree. The model differs from field data in that it predicts a prevalence of generalised individuals of 25–30% whereas existing data for Asturias suggests 16.7%^43^. However, this lower prevalence was derived from a small data set (1 out of 6 being reported as generalised) and recent results from Asturias (personal communication, unpublished findings by the Asturias Government) would now indicate a higher prevalence of generalised in closer agreement with model findings. Also, in areas with wolves the empirical results indicated that cattle TB stayed constant rather than declining. The model results indicated that there would be an increase in wild boar density, a reduction in TB prevalence in wild boar and a reduction in generalised infected wild boar and MTC in the environment, therefore reducing the risk of transmission of MTC to livestock. This can be explained by: firstly, the wildlife reservoir in the Atlantic regions of Spain is composed of two main hosts, wild boar and badger^43^, and wolves are not likely to significantly interfere with badger population dynamics; secondly, the wildlife reservoir contributes to MTC maintenance, but is not the only driver. In Spain, the relative contribution of wildlife to cattle TB breakdowns varies between regions depending on the epidemiological circumstances^62,63^. Cattle movements, for instance, are likely to contribute to TB maintenance^64^.

Our study has highlighted the potential of predation by wolves to reduce TB prevalence in wild boar and thereby reduce the risk of transmission from a key wildlife reservoir of infection. The model framework developed in this study was tailored to the wild boar TB wolf system but the underlying processes that represent the population and epidemiological dynamics are general and therefore we expect the results to apply more broadly. In particular, when predation can regulate a prey species that was previously regulated by virulent pathogens it is likely that infection levels will be reduced. Of course, care must be taken when considering the impact of generalist predators on disease control as they may also prey on alternative species that do not harbour virulent pathogens and therefore where mortality due to predation will not be compensated. Nevertheless, the potential of predators to control infection should be recognised more widely and be contrasted with the detrimental impact of predatory losses to domestic species. The beneficial role of predators should be given more prominence particularly given the need to manage conservation conflicts associated with predator re-establishment^65^.

## Methods

### Ethics statement

All animal sampling took place post-mortem. The wildlife samples were obtained from hunter-harvested individuals that were shot in the legal hunting seasons and independently and prior to our research. According to EU and National legislation (2010/63/UE Directive and Spanish Royal Decree 53/2013) and to the University of Castilla-La Mancha guidelines, no permission or consent is required to conduct the research reported herein.

### Study area and target species

**Figure 5:**
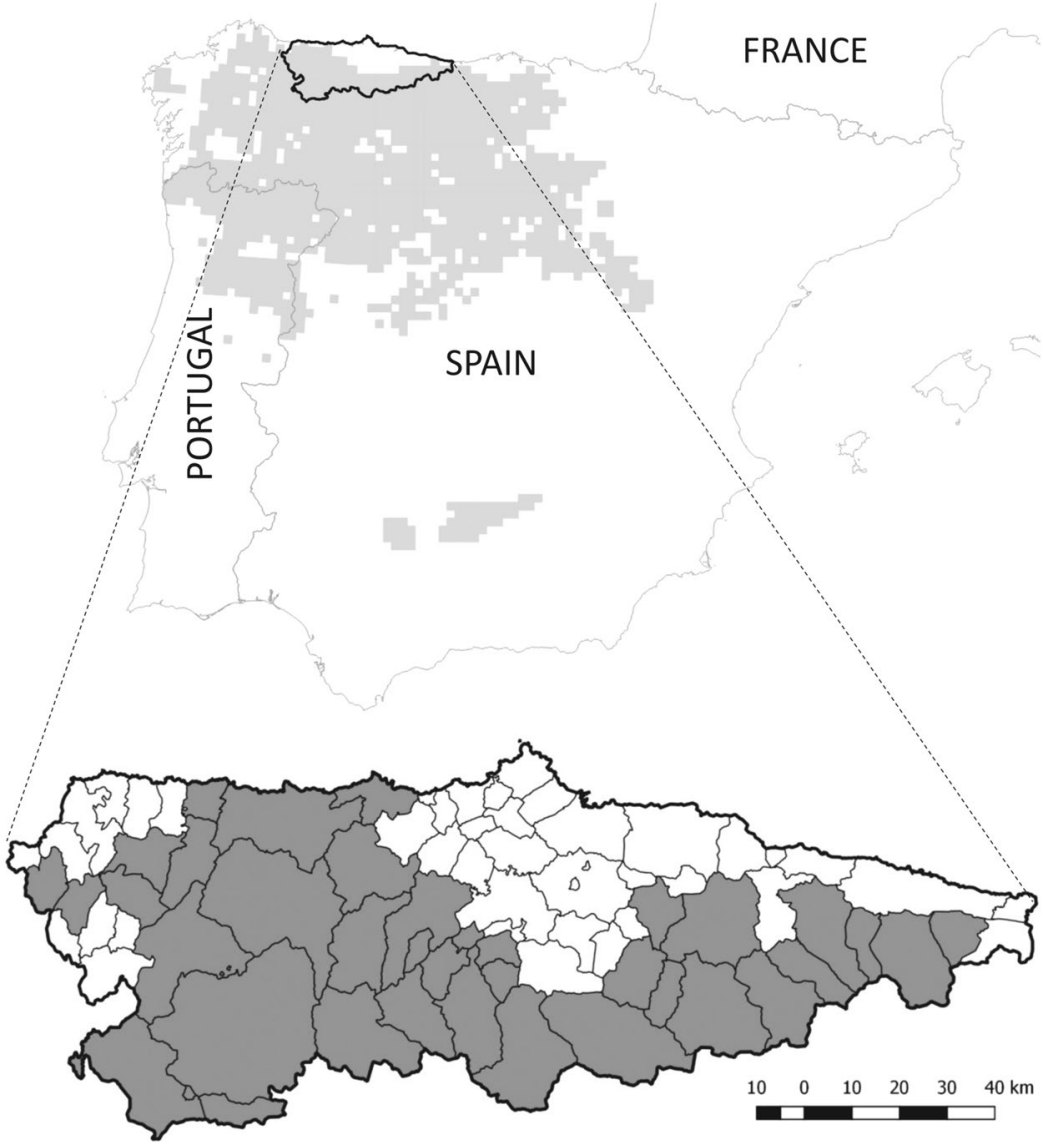
Wolf (*Canis lupus*) distribution maps where the distribution in the Iberian Peninsula is shown in light grey, and municipalities in Asturias, northern Spain, are expanded to show where wolves are present (dark grey) or absent (white).

Asturias, a province of 10,604 km^2^, is located in northwestern Spain (Fig. 5). Wolf population data were obtained from the Asturias Government. Wolf presence is established in two-thirds of Asturias. In the remaining third, containing the majority of coastal regions and the urban and industrial corridors in the centre-north-east of the region (Fig. 5 ^66,67^), wolves are absent or only sporadically recorded. The annual wolf census uses simulated howling and fixed observation points to map wolf packs (methodology detailed in the Asturias Government report^50^) and allows for an estimate of wolf population size^50^. We combine the estimate of wolf abundance for 2003–2004^50^ with data on wolf attack rate on livestock to give a profile of wolf abundance from 2000 to 2014. The regional government also records the number of wild boar harvested on hunting sites annually^51^. Hunting is predominantly non-commercial and traditional among rural inhabitants, taking place in 17 game reserves and 60 municipal hunting estates covering 91% of the province^51^. After standardisation by hunting effort, hunting bag statistics can be used as reliable indices of wild boar relative abundance^68^. We use data describing the temporal variation in the number of wild boar annually hunted (Fig. 1) and in particular generate estimates of wild boar population abundance in 2000–01 and 2013–14.

### TB prevalence

We used serum antibodies against the MTC as an indicator of TB prevalence in wild boar. Serum samples were tested by means of an indirect ELISA using bovine-purified protein derivative (bPPD) following the protocol previously described in Boadella *et al*.^69^. Sample results were expressed as an ELISA percentage (E%) that was calculated using the formula [Sample E% = (sample OD/2 × mean negative control OD) × 100]. Serum samples with E% values greater than 100 were considered positive. Wild boar TB prevalence was available at the municipality level from 2000 to 2014. All cattle herds are tested annually for TB by individual skin testing. This testing is performed and recorded by the Asturias Government. Individual and herd-level data on cattle TB was available from 2005 to 2014, at the municipality scale.

### Asturias: estimating wolf population

We derive an estimate of wolf population abundance for the period 2000–2014 as follows. We use data on wolf population abundance for the period 2003–2004^50^ to obtain an estimate of 252 wolves in Asturias in 2004. We fit a least squares regression on wolf attack rate data for the period 2000–2014 (Fig. S1) to give a rate of increase of wolf abundance. Combining this rate of increase with our wolf abundance estimate for 2004 we estimate that wolf numbers increase linearly from 196 in 2000 to 392 in 2014. Given that both wolf density and wild boar densities are rising and not saturating, and that although wild boar are a preferred prey target for wolves they do not make up the whole wolf diet^33^, we assume that the wolf attack rate on wild boar has not reached saturation levels and therefore model wolf attack rates on wild boar linearly with wolf density.

### Mathematical modelling

We develop a mathematical model that represents the interaction between wild boar, MTC infection and predation. In the model we set disease transmission rates and the wild boar intraspecific competition parameter so the model matches observations for the prevalence of infection and for wild boar density in 2000 and 2014 for the regions of Asturias with wolves. The model findings are extended to consider the areas of Asturias in which wolves are absent, to assess the role of future wolf density in TB control and the potential impact of wolf predation on TB in regions where TB is endemic and prevalence is high.

We separate the population density of wild boar into different age classes to capture distinct disease and reproductive characteristics for piglets (aged 0–1 year) *P*, yearlings (aged 1–2 years) *Y*, and adults (aged 2 years+) *A*. Further, the age-classes are split into susceptible, infected and generalised classes (subscripts *S*, *I*, *G*, respectively) to reflect the disease status of the population. Generalised individuals can also release free-living pathogen with density *F* into the environment. The three different age-classes are required as each class has distinct properties in terms of their demographic and infection dynamics and in the impact of predation. This model framework has been used successfully to understand the impact of vaccination and culling on TB prevalence in the wild boar TB system^28,59^. The model also includes predation by wolves, *W*, and we examine scenarios where wolf density increases from 2000–2014 according to observations and then remains constant or decreases (to represent persecution) thereafter. The population dynamics of the wild boar, TB and wolf system are represented by the following set of non-linear differential equations (which is an extension of classical disease modelling frameworks^70,71^) and of a previous model of wild boar and TB interactions^28,59^.

1a $$\frac{{\rm{d}}{P}_{S}}{{\rm{d}}t}={b}_{A}(Y+A)\,(1-qN)-m{P}_{S}-{d}_{P}{P}_{S}-{\beta }_{DP}{P}_{S}\frac{G}{N}-\omega {\beta }_{FP}{P}_{S}F-{a}_{P}{P}_{S}W$$

1b $$\frac{{\rm{d}}{P}_{I}}{{\rm{d}}t}={\beta }_{DP}{P}_{S}\frac{G}{N}+\omega {\beta }_{FP}{P}_{S}F-m{P}_{I}-{d}_{P}{P}_{I}-{\varepsilon }_{P}{P}_{I}-{a}_{P}{P}_{I}W$$

1c $$\frac{{\rm{d}}{P}_{G}}{{\rm{d}}t}={\varepsilon }_{P}{P}_{I}-m{P}_{G}-\alpha {P}_{G}-{d}_{P}{P}_{G}-{a}_{PG}{P}_{G}W$$

1d $$\frac{{\rm{d}}{Y}_{S}}{{\rm{d}}t}=m{P}_{S}-m{Y}_{S}-{d}_{Y}{Y}_{S}-{\beta }_{DY}{Y}_{S}\frac{G}{N}-\omega {\beta }_{FY}{Y}_{S}F-c{Y}_{S}-{a}_{YA}{Y}_{S}W$$

1e $$\frac{{\rm{d}}{Y}_{I}}{{\rm{d}}t}={\beta }_{DY}{Y}_{S}\frac{G}{N}+\omega {\beta }_{FY}{Y}_{S}F+m{P}_{I}-m{Y}_{I}-{d}_{Y}{Y}_{I}-{\varepsilon }_{Y}{Y}_{I}-c{Y}_{I}-{a}_{YA}{Y}_{I}W$$

1f $$\frac{{\rm{d}}{Y}_{G}}{{\rm{d}}t}={\varepsilon }_{Y}{Y}_{I}+m{P}_{G}-m{Y}_{G}-\alpha {Y}_{G}-{d}_{Y}{Y}_{G}-c{Y}_{G}-{a}_{G}{Y}_{G}W$$

1g $$\frac{{\rm{d}}{A}_{S}}{{\rm{d}}t}=m{Y}_{S}-{d}_{A}{A}_{S}-{\beta }_{DA}{A}_{S}\frac{G}{N}-\omega {\beta }_{FA}{A}_{S}F-c{A}_{S}-{a}_{YA}{A}_{S}W$$

1h $$\frac{{\rm{d}}{A}_{I}}{{\rm{d}}t}={\beta }_{DA}{A}_{S}\frac{G}{N}+\omega {\beta }_{FA}{A}_{S}F+m{Y}_{I}-{d}_{A}{A}_{I}-{\varepsilon }_{A}{A}_{I}-c{A}_{I}-{a}_{YA}{A}_{I}W$$

1i $$\frac{{\rm{d}}{A}_{G}}{{\rm{d}}t}={\varepsilon }_{A}{A}_{I}+m{Y}_{G}-\alpha {A}_{G}-{d}_{A}{A}_{G}-c{A}_{G}-{a}_{G}{A}_{G}W$$

1j $$W=W(t)$$

Here, $N=P+Y+A$ represents the total wild boar population where $P={P}_{S}+{P}_{I}+{P}_{G}$, $Y={Y}_{S}+{Y}_{I}+{Y}_{G}$, $A={A}_{S}+{A}_{I}+{A}_{G}$ and *G* is the total number of generalised, $G={P}_{G}+{Y}_{G}+{A}_{G}$. Susceptible and infected yearlings and adults give birth to susceptible piglets at rates *b*_*Y*_ and *b*_*A*_ respectively. Generalised yearlings and adults give birth to piglets at rate *b*_*G*_. Here we assume that ${b}_{A}={b}_{Y}={b}_{G}$. The total population is regulated through a crowding parameter, *q*, that acts on the birth rate. Maturity from piglets to yearlings and yearlings to adults occurs at rate *m* and piglets, yearlings and adults may die of natural causes at rates *d*_*P*_, *d*_*Y*_, *d*_*A*_ respectively. Here we assume *d*_*P*_ = *d*_*Y*_ = *d*_*A*_. This set-up for the demographic dynamics has previously been used to assess wild boar TB interactions^28,59^.

We assume infection can occur through direct frequency-dependent interactions (since wild boar tend to congregate in social groups) between susceptible and generalised individuals with transmission coefficients *β*_*DP*_, *β*_*DY*_ and *β*_*DA*_ or through environmental contact with free-living MTC, with transmission coefficients *β*_*FP*_, *β*_*FY*_ and *β*_*FA*_ for the different age classes respectively. Piglets and yearlings are more likely to become infected through both direct and environmental infection than adults^58^, and we assume that they are three times more susceptible to infection than adults to ensure that the model presents sufficient levels of infection in yearlings as compared to adults. In this way we have set the model so that the yearling class is the same as the piglet class in terms of disease characteristics, but the yearling class is the same as the adult class in terms of reproductive processes. Infected individuals are not infectious but can progress to the generalised (infectious) class at rates ${\varepsilon }_{P}$, ${\varepsilon }_{Y}$ and ${\varepsilon }_{A}$. In Asturias where resources are not limited we assume ${\varepsilon }_{P}={\varepsilon }_{Y}={\varepsilon }_{A}$. Later we consider regions where resources (particularly water) are scarce and overall health is impaired (similar to conditions in central and southern Spain). Co-infection can lead to a greater risk of becoming a super-shedder^72^ and we assume that piglets and yearlings progress from the infected to the generalised class at three times the rate of adults (${\varepsilon }_{P}={\varepsilon }_{Y}=3{\varepsilon }_{A}$). We assume that free-living MTC is shed from generalised wild boar at rate *λ* and decays at rate *μ*. The level of environmental transmission is scaled through the parameter $\omega$ which increases when environmental conditions become more severe to reflect, for example, aggregation at limited water holes ($\omega =0.1$ in Asturias and $\omega =1$ in resource limited regions).

We assume that wild boar suffer mortality, in addition to natural death, from three causes: individuals in the generalised class suffer an additional disease induced mortality at rate *α*; all adult and yearling classes are culled due to hunting at constant rate *c*; and wolves successfully prey on susceptible and infected piglets at rate *a*_*P*_, generalised piglets at rate *a*_*PG*_, generalised yearlings and adults at rate *a*_*G*_ and susceptible and infected yearlings and adults at rate *a*_*YA*_. Our baseline assumption is that ${a}_{YA}=0$ and ${a}_{P}={a}_{PG}={a}_{G}$ implying that wolves prey on piglets and generalised individuals only (although we do consider alternative predation assumptions). Further parameter description and the parameter values used in this study are shown in Supplementary Information.

## Supplementary information

Supplementary Information

## Data Availability

A reporting summary for this article is available in the Supplementary Information. The supporting MATLAB code to reproduce Figs 2–4 and S3–S5 will be deposited in an external repository on acceptance.
