# Supplementary material for: Wolves contribute to disease control in a multi-host system
Source: Sci Rep. 2019 May 28;9:7940. doi: 10.1038/s41598-019-44148-9 (PMC6538665; doi:10.1038/s41598-019-44148-9)
Supplement: Supplementary file 1 — Supplementary Information [file 41598_2019_44148_MOESM1_ESM.pdf]

Supplementary Information to “Wolves contribute to disease control in a multi-host system”

Eleanor Tanner, Andy White, Pelayo Acevedo, Ana Balseiro, Jaime Marcos and Christian Gortázar

Wild boar TB model parameters

The parameters for wolf wild boar TB model[1,2] are as follows. Note that where there are two values indicated, the first value represents the parameter value for Asturias, whereas the second value represents the parameter value for areas of high TB prevalence, for example southern Spain.

$b_A = \log(4)$ The population birth rate in a disease-free population when resources are unlimited. This constant rate means that for each reproductive member of the population, 3 piglets will be born, averaged over the population over a year. (This has been derived by assuming that there is a 50% sex ratio and that each female produces an average of 6 offspring per year when resources are not limited.) Units: $year^{-1}$.

$K = 4.59$ (Asturias); 72.78 (high prevalence region) The carrying capacity for the total population density in the absence of disease: in Asturias, set to 4.59; in a high prevalence region where wild boar live at higher density and disease is more prevalent this is set to 72.78. Units: *population density*.

$q = \frac{1}{K}\left(1 - \frac{d_A(d_P+m)(d_Y+m)}{m(b_A m + b_Y d_A)}\right)$ This parameter limits the total population to the carrying capacity K in the populated disease-free steady state, and is derived from steady-state analysis of the model without infection. Units: $density^{-1}$.

$m = 1$ The rate that piglets mature to yearlings and yearlings mature to adults. These rates assume that it takes on average 1 year to enter the next age class. Units: $year^{-1}$.

$d_P = d_Y = d_A = \frac{1}{7}$ The natural death rate of all classes which implies an average life expectancy of 7 years. Units: $year^{-1}$.

$c = 0.3$ The continuous culling rate effective on all yearlings and adults, set to achieve a total hunting bag of approximately 20% of the wild boar population in Asturias. Units: $year^{-1}$.

$\beta_{DA} = 1.06$ The rate that adults are infected by direct contact frequency dependent transmission fitted to give prevalence levels observed in the wild boar TB system in Asturias. Units: $year^{-1}$.

$\beta_{DP} = \beta_{DY} = c_\beta \beta_{DA}$ The direct contact frequency dependent infection rate for piglets and yearlings. We assume that $c_\beta = 3$ and so disease transmission to piglets and yearlings is three times that of the adult rate. Units: $year^{-1}$.

$\beta_{FA} = \frac{20}{K}$ The rate that adults are infected by contact through environmental infection fitted to give prevalence levels observed in the wild boar TB system in Asturias. Units: $density^{-1} \times year^{-1}$

$\beta_{FP} = \beta_{FY} = c_\beta \beta_{FA}$ The environmental infection rate for piglets and yearlings. We assume that $c_\beta = 3$ and so disease transmission to piglets and yearlings is three times that of the adult rate. Units: $density^{-1} \times year^{-1}$.

$\omega = 0.1$ (Asturias); 1 (high prevalence regions) This parameter scales the level of environmental transmission. Environmental transmission typically occurs at shared water holes and it therefore high when water resources are scarce. In Asturias $\omega = 0.1$ to reflect plentiful resources. In high prevalence regions (such as central and southern Spain) resources are scarce and $\omega = 1$. Units: *scalar*.

$\varepsilon_A = \frac{2}{3}$ This is the rate that infectious adults become generalised. This assumes that it takes on average 18 months for an infected adult to progress to the generalised class. Units: $year^{-1}$.

$\varepsilon_P = \varepsilon_Y = c_\varepsilon \varepsilon_A$ The rate that infected piglets and yearlings become generalised. In the Asturias model these are set to the same value as that for adults ($c_\varepsilon = 1$). For high prevalence regions $c_\varepsilon = 3$ assuming that it takes on average 6 months for an infected piglet or yearling to progress to the generalised class. This value is 3 times that of adults, and recognises the fact that in high prevalence regions that typically have scarce resources the body condition of wild boar is generally poor and co-infections are common. It is acknowledged that the stress of co-infections can shorten the length of time to progress to generalised[3]. Units: $year^{-1}$.

$\alpha = 1$ This is the additional disease induced death rate of the generalised class and assumes that on average individuals spend 1 year in the generalised class before death. Units: $year^{-1}$.

$\lambda = 1$ The rate of shedding of infectious particles by generalised classes. We normalise this value to 1. This is valid as we have explored a range of values for β_P, β_Y and β_A which scale with the size of λ and the density of free-particles, F. Units: $year^{-1}$.

$\mu = 6$ This is the decay rate for free-living particles, indicating that they have an average life expectancy of 2 months. Units: $year^{-1}$.

$a_P = a_{PG} = a_{YG} = a_{AG} = 0.00099$ The successful predation rates for wolves on susceptible and infected piglets, generalised piglets, generalised yearlings and generalised adults respectively following Nores *et al.* 2008[4]. Units: $(wolf\ density)^{-1} \times year^{-1}$.

Asturias: prey selection

Figures S3 and S4 represent the same scenario as in main paper Figure 2 for the period 2000-2014 except for changes to the classes of wild boar that wolves prey on. In both these cases wolf predation, measured as the number of wild boar taken per year per wolf, is adjusted to be similar over the first 10 years to that of main paper Figure 2. This results in using the same predation rate when only piglet predation occurs, and a halved predation rate for indiscriminate predation.

Figure S3 shows that when wolves prey indiscriminately, infected prevalence is reduced but not as quickly as in main paper Figure 2 for the period 2000-2014. However, as now there is also predation on adult and yearling wild boar, there is less reproduction and therefore a reduction in the overall population density and the number of susceptibles available for infection. This results in a 40% decrease in the level of the pathogen in the environment, and also reduced wild boar density.

Figure S4 shows that when wolves only prey on piglets, infection is not reduced as quickly as for predation on piglets and generalised individuals (main paper Figure 2) such that the number of generalised wild boar in the population and hence the level of free living pathogen is not significantly reduced. The wild boar density rises, but the disease prevalence is not reduced significantly.

References

1. Díez-Delgado, I. *et al.* Impact of piglet oral vaccination against tuberculosis in endemic free-ranging wild boar populations. *Prev. veterinary medicine* **155**, 11–20 (2018).
2. Tanner, E. *et al.* The critical role of infectious disease in compensatory population growth in response to culling. *The Am. Nat.* (in press 2019).
3. Lass, S. *et al.* Generating super-shedders: co-infection increases bacterial load and egg production of a gastrointestinal helminth. *J. Royal Soc. Interface* **10**, 20120588 (2013).
4. Nores, C., Llaneza, L. & Álvarez, A. Wild boar sus scrofa mortality by hunting and wolf canis lupus predation: an example in northern spain. *Wildl. biology* **14**, 44–51 (2008).

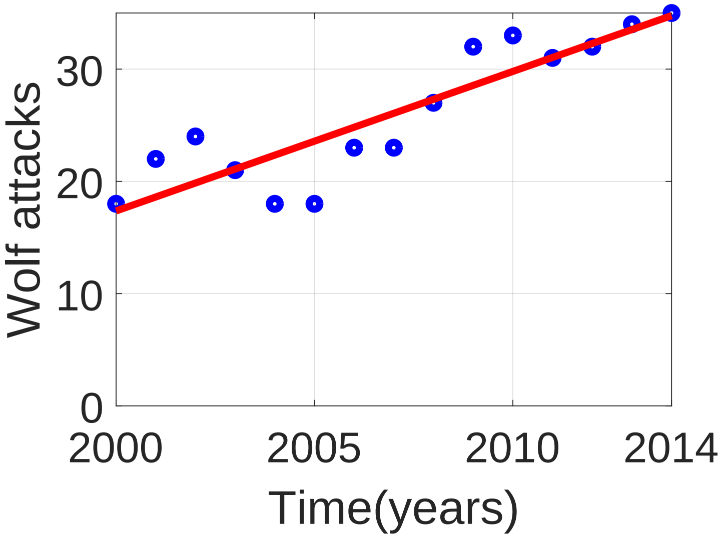

Figure S1. Asturias: the rate of increase in wolf attacks. Rate of increase in Asturias wolf attacks for 2000-2014 derived from least-squares linear regression on wolf attack data, Asturias government data. Blue spots show the annual wolf attack data count. The red line is the least-squares linear regression of the wolf attack data.

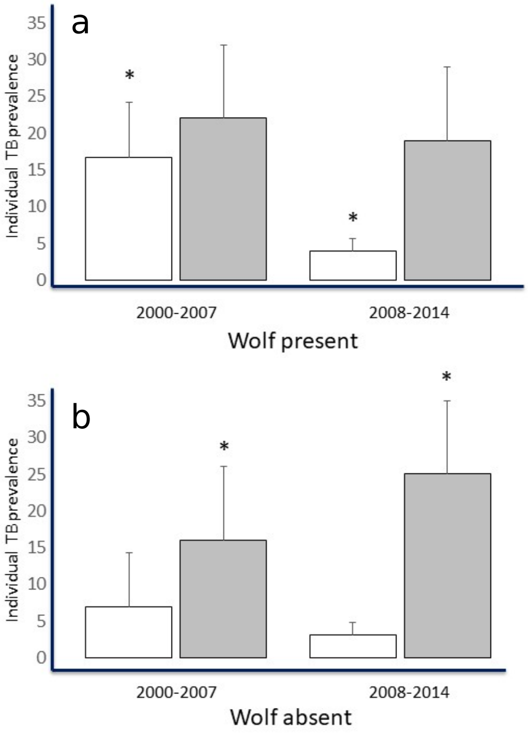

Figure S2. Asturias: mean TB prevalence for wild boar and cattle. Mean individual TB prevalence for wild boar (serum antibodies; white columns) and cattle ($\times$100; skin test reactors; grey columns) in Asturias. The upper panel (a) represents areas where wolves are present and the lower one (b) those where wolves are absent. Cattle data were only available for the period between 2005 and 2014. Bars represent 95% confidence intervals and asterisks indicate significant differences at $p<0.01$.

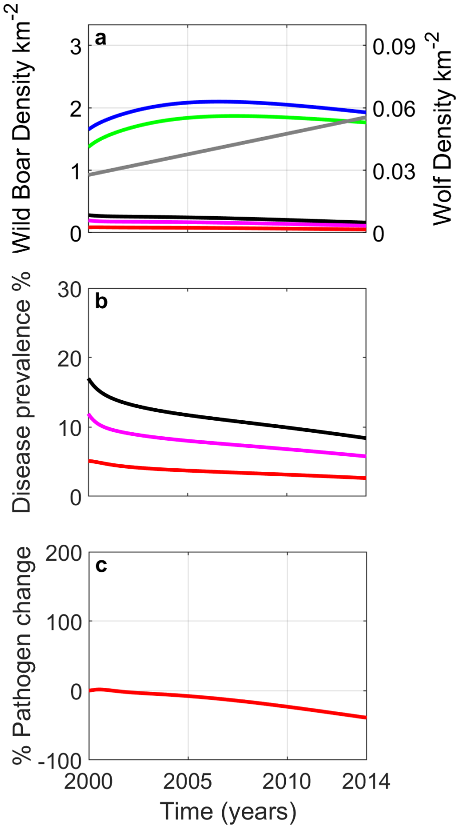

Figure S3. Asturias: effect of wolves preying on all classes of wild boar. The effect on (a) wild boar density, (b) TB prevalence, (c) free-living density when predation by wolves on all classes of wild boar. For parameter values see Supplementary Information.

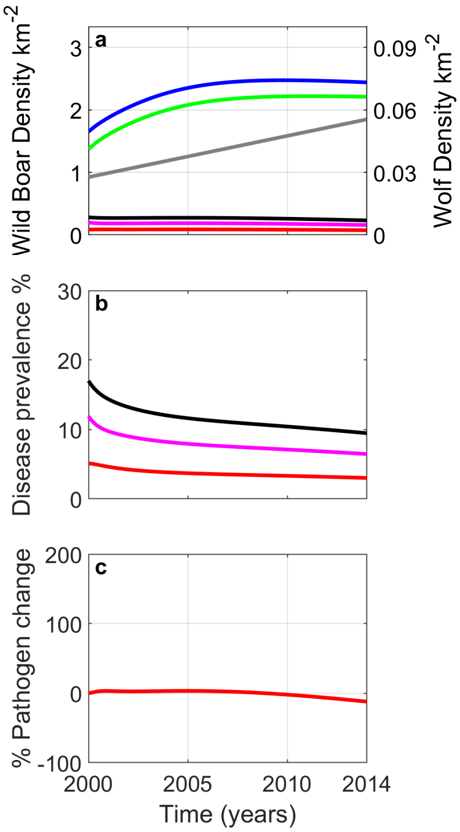

Figure S4. Asturias: effect of wolves preying on wild boar piglets only. The effect on (a) wild boar density, (b) TB prevalence, (c) free-living density when predation by wolves in on wild boar piglets only. For parameter values see Supplementary Information.

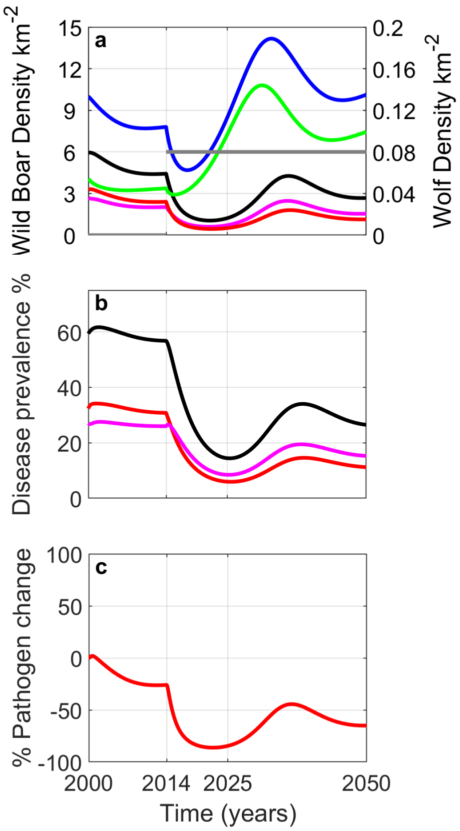

Figure S5. High TB prevalence area: effect of wolves preying on wild boar. The effect on (a) wild boar density, (b) TB prevalence, (c) free-living density after a constant density of wolves (0.08/km^2) is introduced to a wild boar population with density steady at 8/km^2 and disease prevalence ($(I+G)/N$) of 57%. Wolves prey on wild boar at the same rate as in Asturias. For parameter values see Supplementary Information.
